# Neoadjuvant Immunotherapy for MSI-H/dMMR Locally Advanced Colorectal Cancer: New Strategies and Unveiled Opportunities

**DOI:** 10.3389/fimmu.2022.795972

**Published:** 2022-03-17

**Authors:** Xuan Zhang, Tao Wu, Xinyi Cai, Jianhua Dong, Cuifeng Xia, Yongchun Zhou, Rong Ding, Renfang Yang, Jing Tan, Lijuan Zhang, Ya Zhang, Yuqin Wang, Chao Dong, Yunfeng Li

**Affiliations:** ^1^ Department of Colorectal Surgery, Yunnan Cancer Hospital, The Third Affiliated Hospital of Kunming Medical University, Kunming, China; ^2^ Laboratory of Molecular Diagnosis Center, Yunnan Cancer Hospital, The Third Affiliated Hospital of Kunming Medical University, Kunming, China; ^3^ Department of Minimally Invasive Intervention, Yunnan Cancer Hospital, The Third Affiliated Hospital of Kunming Medical University, Kunming, China; ^4^ Department of Imaging, Yunnan Cancer Hospital, The Third Affiliated Hospital of Kunming Medical University, Kunming, China; ^5^ Department of Pathology, Yunnan Cancer Hospital, The Third Affiliated Hospital of Kunming Medical University, Kunming, China; ^6^ Department of Oncology, Yunnan Cancer Hospital, The Third Affiliated Hospital of Kunming Medical University, Kunming, China

**Keywords:** locally advanced colorectal cancer, neoadjuvant therapy, immunotherapy, mismatch repair deficiency, microsatellite instability-high

## Abstract

Patients with locally advanced colorectal cancer (LACRC) have a high risk of recurrence and metastasis, although neoadjuvant therapy may provide some benefit. However, patients with high microsatellite instability/deficient mismatch repair (MSI-H/dMMR) LACRC receive little benefit from neoadjuvant chemoradiotherapy (nCRT) or neoadjuvant chemotherapy (nCT). The 2015 KEYNOTE-016 trial identified MSI-H/dMMR as a biomarker indicative of immunotherapy efficacy, and pointed to the potential use of immune checkpoint inhibitors (ICIs). In 2017, the FDA approved two ICIs (pembrolizumab and nivolumab) for treatment of MSI-H/dMMR metastatic CRC (mCRC). In 2018, the CheckMate-142 trial demonstrated successful treatment of mCRC based on “double immunity” provided by nivolumab with ipilimumab, a regimen that may become a standard first-line treatment for MSI-H mCRC. In 2018, the FDA approved nivolumab alone or with ipilimumab for patients who progressed to MSI-H/dMMR mCRC after standard chemotherapy. The FDA then approved pembrolizumab alone as a first-line treatment for patients with MSI-H/dMMR CRC that was unresectable or metastatic. There is now interest in using these drugs in neoadjuvant immunotherapy (nIT) for patients with MSI-H/dMMR non-mCRC. In 2020, the NICHE trial marked the start of using nIT for CRC. This novel treatment of MSI-H/dMMR LACRC may change the approaches used for neoadjuvant therapy of other cancers. Our review of immunotherapy for CRC covers diagnosis and treatment, clinical prognostic characteristics, the mechanism of nIT, analysis of completed prospective and retrospective studies, and ongoing clinical trials, and the clinical practice of using nIT for MSI-H/dMMR LACRC. Our team also proposes a new organ-preservation strategy for patients with MSI-H/dMMR low LARC.

## Introduction

Global cancer statistics for 2020 indicated that colorectal cancer (CRC) is the most common malignant tumor of the digestive system, and that CRC ranked third in incidence rate and second in mortality rate among all cancers (1). The increases in industrialization and urbanization and in the number of elderly have contributed to the significantly increasing incidence rate and mortality rate of CRC during recent years. Most CRC patients are in the stage of local progression upon diagnosis, making effective treatment more difficult.

Locally advanced colorectal cancer (LACRC) is defined as CRC stage II (cT3–4, N0)/stage III (any cT, N+). During recent years, advancements in standardized surgery and subsequent improvements in neoadjuvant therapy have improved outcomes. The advantages of neoadjuvant therapy are that it can reduce tumor stage, improve the rate of R0 resection, decrease the rate of local recurrence, and enable some patients to achieve a clinical complete response (cCR) or even a pathological complete response (pCR) (2). However, distant metastases, surgical morbidities, and adverse effects (AEs) caused by neoadjuvant radiotherapy or chemotherapy remain significant problems.

With the advent of the era of precision medicine, researchers have begun to explore the effect of microsatellite status on the characteristics of tumors (3). However, high microsatellite instability/defective mismatch repair (MSI-H/dMMR) LACRC has low sensitivity to current neoadjuvant therapies. Fortunately, the emergence of neoadjuvant immunotherapy (nIT) brings new hope to these patients (4). It is necessary to examine the suitability of nIT for patients with MSI-H/dMMR locally advanced rectal cancer (LARC). Therefore, this review comprehensively summarizes the new strategies and opportunities that nIT may provide for these patients.

## Current Dilemmas in Treatment of Locally Advanced Colorectal Cancer

LACRC has a high risk of postoperative local recurrence and distant metastasis. The traditional mode of surgical resection cannot meet the needs of all of these patients, although neoadjuvant therapy can sometimes provide additional efficacy. What is the efficacy of neoadjuvant chemotherapy (nCT) for LACRC patients with MSI-H/dMMR? Subgroup analysis of data in the FOxTROT study, which was reported at the 2019 ASCO Annual Meeting, indicated that the effective rate of nCT for CRC in patients with MSI-H/dMMR was only 4.7% (5). Among all patients, 73.6% had tumor regression based on tumor pathological examination, but only 26.6% who had proficient mismatch repair/microsatellite stability (pMMR/MSS) had no tumor regression (5). A 2020 article in Clinical Cancer Research described the use of FOLFOX as a nCT regimen for rectal cancer in a trial conducted at the Memorial Sloan Kettering Cancer Center. The results indicated that tumor progression occurred in 29% of patients in the MSI-H/dMMR group, but in none of the patients in the pMMR group (6). These two studies thus illustrated that most CRC patients with MSI-H/dMMR do not benefit from the FOLFOX nCT regimen.

For patients with LARC, the National Comprehensive Cancer Network (NCCN) guidelines recommend “neoadjuvant chemoradiotherapy (nCRT) + total mesorectal excision + adjuvant chemotherapy”, and this is generally recognized as a standard treatment scheme worldwide (7). Nevertheless, there are some different views regarding the efficacy of nCRT in these patients. First, although nCRT for rectal cancer reduces the local recurrence rate, it does not improve the long-term survival rate of patients. Second, nCRT for rectal cancer can lead to post-surgical complications, such as anastomotic leakage and poor healing of the perineal wound, as well as long-term toxicities, such as bladder dysfunction, loss of anal sphincter function, and sexual dysfunction. Finally, about 20% of LACRC patients are insensitive to nCRT, and if the tumor progresses after nCRT, these patients may miss the opportunity of radical operation. A retrospective study in the United States National Cancer Data Base indicated that the postoperative pCR rate of LACRC patients after nCRT was 8.9% in an MSS group and 5.9% in an MSI-H group (P = 0.01). These results suggested that LACRC patients with MSI-H were less sensitive to radiotherapy and chemotherapy (8).

Treatments for CRC have continued to improve. Molecular genotyping and determination of microsatellite status were significant contributions to the development of precision therapies (9). In recent years, immune checkpoint inhibitors

(ICIs), antibodies that target PD-1, PD-L1, or CTLA-4, have emerged as effective treatments for a variety of cancers (10). The benefits provided by these ICIs even surpass those provided by targeted therapy in some aspects, and ICIs may also provide an improved treatment for CRC (11). Thus, although CRC patients with MSI-H status may have poor prognoses, they also uniquely benefit from treatment with ICIs.

## Significance of MSI-H/dMMR in Tumors

MSI refers to variation of MS sequence length or base composition due to insertion or deletion mutations during DNA replication, an alteration often caused by dMMR. The MS status of a tumor may be classified as stable (MSS), or as having low instability (MSI-L) or high instability (MSI-H) (12). MSI-H has a high incidence in solid tumors, such as endometrial cancer, CRC, and gastric cancer (13). dMMR can be due to a germline mutation of the MMR gene (Lynch syndrome), in which case there is a hereditary pattern of malignant tumors (14). However, dMMR more commonly occurs from sporadic mutations of the MMR gene, a condition usually accompanied by the CpG island methylation phenotype (CIMP). Notably, 50% of the sporadic cases also exhibit a BRAF-activating mutation (V600E). Thus, Lynch syndrome can be usually excluded in patients with CIMP and this BRAF mutation (15, 16). The clinical presentations of patients with dMMR and MSI-H are indistinguishable, and their detection rates are consistently very high. Although these two aberrations are often not distinguished in the clinic, they are not identical (17). Most clinical studies demonstrated that patients with MSI-H/dMMR solid tumors received obvious benefits from ICIs (18–20). Moreover, a key factor determining the benefit of ICI therapy is the MSI-H status of the patient, not the specific kind of cancer (21).

In 2017, the United States FDA approved pembrolizumab (anti-PD-1 antibody) for the treatment of patients with advanced MSI-H/dMMR or metastatic solid tumors. These patients typically experience disease progression following standard treatment, and no other satisfactory alternative treatments are available. This is the first approved anti-tumor therapy that considered specific biomarkers rather than the type of tumor. Since then, MSI status has become an important consideration in clinical oncology. Because of the importance of MSI-H/dMMR status in predicting response to tumor immunotherapy, many guidelines recommend determination of the MSI status of patients with advanced solid tumors before administering immunotherapy (22).

## Unique Prognostic Characteristics of MSI-H/dMMR CRC

MSI-H/dMMR CRC is a unique type of CRC defined by biomarker status that is present in 12 to 15% of all CRC patients, and is more likely to occur in those with right-sided cancers (i.e., poorly differentiated and/or mucinous adenocarcinoma). Only 2% of patients with rectal cancer are MSI-H (18). There is also evidence that the MS status of CRC changes during cancer progression. In particular, patients with late-stage cancers are less likely to be MSI-H (23); patients with early-stage MSI-H/dMMR CRC have good prognoses, but this status is considered a poor prognostic factor in patients with metastatic CRC (mCRC) (24).

Patients with MSI-H/dMMR locally advanced colon cancer (LACC) have unique clinicopathological and molecular biological characteristics, and typically experience slow clinical progression. These patients are less likely to experience lymph node metastasis and distant metastasis. Patients with MSI-H/dMMR stage II CRC generally have good prognoses. Following surgery alone, these patients have a 5-year survival rate as high as 80%, and fluorouracil alone provides little additional benefit. Thus, the present consensus is that adjuvant chemotherapy is not needed for patients with low-risk stage II CRC after surgery. However, patients with MSI-H/dMMR stage III CRC who received postoperative adjuvant chemotherapy have better prognoses than MSS patients. Moreover, the efficacy of oxaliplatin adjuvant therapy appeared to be unaffected by MSI/MMR status (25, 26).

Patients with MSI-H/dMMR stage IV CRC account for about 4 to 5% of all CRC patients (18). A summary analysis of four clinical studies on first-line treatment for mCRC indicated that the median overall survival (OS) was 13.6 months for patients with dMMR and 16.8 months for patients with pMMR (HR = 1.35; 95% CI: 1.13~1.61; P = 0.001) (27). The prognosis of patients with MSI-H/dMMR mCRC is poor, especially in those with the BRAF V600E mutation (28).

A 2016 study revealed that the levels of cytotoxic cells, CD8+, Th1, Th2, follicular helper T cells, and T cell markers were significantly higher in patients with MSI-H/dMMR CRC than in those with MSS/pMMR CRC (24). Another 2019 study reported that patients with MSI-H/dMMR CRC had a higher tumor mutation burden (TMB), tumor neoantigen burden (TNB), and greater lymphocyte infiltration of tumor tissues. Notably, these patients also had increased expression of PD-L1 in the tumor microenvironment (TME), suggesting that ICIs have potential for treatment of these individuals (29).

## Current Status of Immunotherapy for MSI-H/dMMR CRC

The latest NCCN guidelines (v1.2021) for gastric cancer (30), colon cancer (31) and rectal cancer (32) revised the previous recommendations for detection of MSI/MMR status. These new guidelines recommend that all newly diagnosed patients should be tested for MSI using the polymerase chain reaction and for MMR using immunohistochemistry (IHC). The previous recommendations limited this testing to patients with suspected metastasis. The new guidelines therefore mean that ICIs are not only suitable for rescue treatment of patients with MSI-H/dMMR stage IV CRC, but also as nIT for patients with earlier stages of CRC.

For patients who have unresectable advanced or mCRC and are suitable for high-intensity chemotherapy, the v1.2021 NCCN guidelines recommend nivolumab ± ipilimumab (O ± Y) or pembrolizumab as a first-line treatment for patients with MSI-H/dMMR status (32, 31). These guidelines recommend pembrolizumab as the preferred treatment, indicating that immunotherapy now has priority over traditional chemotherapy ± targeted therapy.

In contrast to the 2020 NCCN guidelines for colon cancer, the newest guidelines added nivolumab ± ipilimumab or pembrolizumab (preferred) as adjuvant treatment for patients with MSI-H/dMMR CRC (31) — a new immune adjuvant treatment scheme for these patients. In addition, the newest guidelines recommend nivolumab ± ipilimumab or pembrolizumab (preferred) as an option for preoperative neoadjuvant therapy for resectable MSI-H/dMMR mCRC (31, 32). This is the first time the NCCN recommended an immunotherapy as a preoperative neoadjuvant therapy for CRC. However, the NCCN has not yet provided guidelines regarding the use of nIT for LACRC.

## Anti-Tumor Mechanism of nIT

The most widely studied ICIs are antibodies that target CTLA-4, PD-1, and PD-L1, and several ICIs are approved for different types of cancers. PD-L1 binds to PD-1, and this immune checkpoint pathway has a key role in inhibiting T cell-mediated antitumor immune response due to the interaction of PD-1 and CD80 on T cells in the TME. PD-1 and PD-L1 inhibitors inhibit the activity of this immune checkpoint, effectively “releasing the immune brake” in the TME by inducing T cell activation, reactivating the immune response of T cells on tumors, and increasing immune system function (33, 34). The aim of traditional nCT is to reduce tumor staging, whereas nIT aims to enhance the body’s general immunity to tumor antigens. Patients with early- and middle-stage tumors have relatively sound immune systems, and their tumor burdens are generally not as severe. These patients express many new tumor antigens before operation, and this can increase the activity of anti-tumor immune T cells, followed by dispersal throughout the body and removal of micro-metastases. After a multi-line treatment, the physical and immune state of these patients has various degrees of dysfunction. Therefore, a better response could theoretically be achieved by earlier application of immunotherapy.

**Figure 1:**
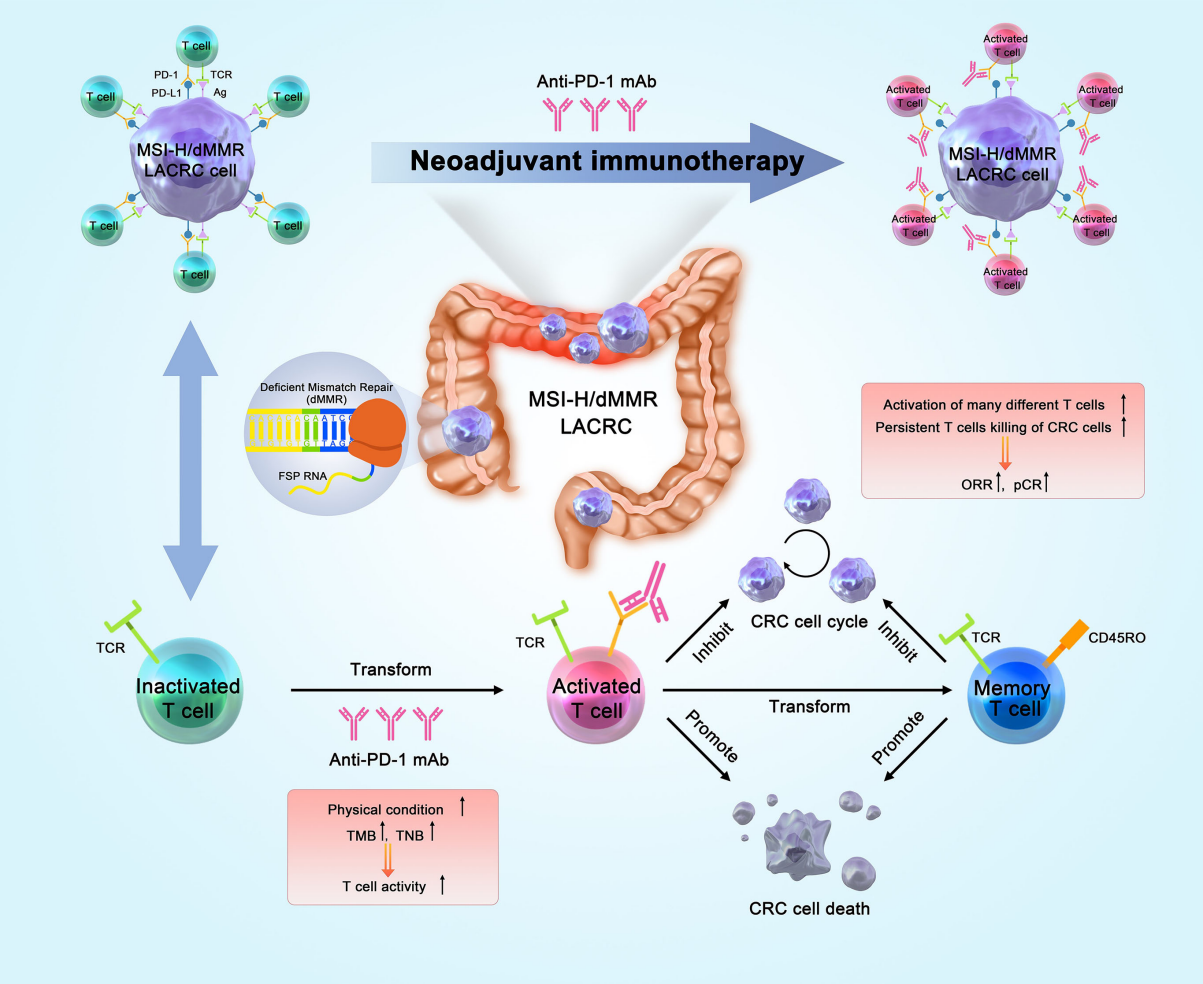
Anti-tumor mechanism of nIT in patients with MSI-H/dMMR CRC. nIT, Neoadjuvant immunotherapy; MSI-H, Microsatellite instability-high; dMMR, Deficient mismatch repair; CRC, Colorectal cancer; TMB, Tumor mutation burden; TNB, Tumor neo-antigen; ORR, Objective response rate; pCR, Pathological complete response.

The basis of nIT is that it induces the expansion of T cells during the early stage of cancer and leads to less impairment of T cell function by targeting the elevated level of endogenous tumor antigens in the primary tumor. Thereafter, nIT can kill tumor cells, eliminate micro-metastases, promote preoperative down-staging, improve the rates of R0 resection and pathological remission, and reduce the rate of postoperative recurrence (35) (**Figure 1**).

Studies of animal models indicated that nIT produced more tumor-related CD8+ effector T cells in the peripheral blood and organs. The survival time of most mice with high levels of CD8+T cells was more than 100 days. Even after tumor clearance, the number of specific CD8+T cells remained stable. This suggests that nIT may induce the immune system to continue killing tumor cells, thus providing a long-term and robust response (36).

## Prospective Clinical Trials of nIT for MSI-H/dMMR Non-mCRC

The discovery of MSI-H/dMMR as a potential biomarker for CRC immunotherapy greatly stimulated research in this area. There has been extensive investigation of the effect of anti-PD-1/anti-PD-L1 therapy on non-mCRC, and there are many encouraging preliminary results (37). Below, we summarize the recent clinical progress in the use of nIT for LACRC to illustrate the potential of nIT for these patients (**Table 1**).

**Table 1:** Prospective clinical trials of nIT for MSI-H/dMMR non-mCRC.

Study	Year	Country	Cases	Location	Stage	Neoadjuvant therapy strategy	Efficacy	AEs
NICHE (Phase II)	2020	Netherlands	dMMR: 20 pMMR: 15	Colon cancer	I-III	dMMR/pMMR: Ipilimumab (1mg/kg Day 1); Nivolumab (3mg/kg Day 1+15) → surgery pMMR: Ipilimumab (1mg/kg Day 1); Nivolumab (3mg/kg Day 1+15); Celecoxib (200mg daily) → surgery	pathologic response: dMMR vs pMMR = 100%(20/20) vs 27%(4/15); MPR: dMMR vs pMMR = 95%(19/20) vs 20%(3/15); pCR in dMMR = 60%(12/20)	Grade 3-4 immunotherapy related AEs =13%; Surgery related complications =10%
NICOLE (Phase II)	2021	/	Nivolumab group: 22 (MSI-H vs MSS=3 vs 19) Control group: 22 (MSI-H vs MSS=5 vs 17)	Colon cancer	II-III	Nivolumab group: nivolumab 2cycles→ surgery; control group: surgery	Nivolumab group: R0 resection rate = 100%; Down-staging rate = 70%	Nivolumab group: Delay or surgical complications = 0 ; Grade 3 diarrhea = 4.5%(1/22)
VOLTAGE-A (Phase II)	2020	Japan	MSS: 37 (A1 group) MSI-H: 5 (A2 group)	Rectal cancer	II-III	nCRT(50.4Gy+Cape)→Those who did not progress were enrolled→ 5 courses of nivolumab in the intermittent period→ Surgery→ Adjuvant chemotherapy with FOLFOX or XELOX	MSS: MPR=38%(14/37), pCR=30%(11/37); 1 case adapted W&W strategy after achieving cCR; MSI-H: pCR = 60%(3/5)	Grade 3 myasthenia, interstitial nephritis and grade 2 peripheral motor neuropathy occurred, but they could be relieved without affecting the subsequent surgery
NRG-GI002 (Phase II)	2021	America	Control group: 68 Pembrolizumab group: 69	Rectal cancer	II-III	FOLFOX * 4months→ nCRT(50.4Gy+Cape) +/-Pembrolizumab (200mg q3w*6cycles) → Surgery (8~12 weeks after radiotherapy)	control group vs pembrolizumab group: NAR score: 14.08 vs 11.53 (p=0.26); pCR: 29.4% vs 31.9%(p=0.75); cCR: 13.6% vs 13.9%(p=0.95); Sphincter preserving surgery: 71.0% vs 59.4%(p=0.15)	Grade 3-4 AEs in Pembrolizumab group: During nCRT vs after nIT = 48.2% vs 37.3%
NCT04231552 (Phase II)	2021	China	pMMR: 26 dMMR: 1	Rectal cancer	II-III	SCRT(5x5Gy) → One week later, Capox + Carrelizumab (200mg q3w*2cycles) → Surgery after 1 week	pCR: 48.1% [MSS vs MSI-H = 46%(12/26) vs100%(1/1)]; Down-staging rate: 70%; Anus preserving rate: 89%; R0 resection rate: 100%	Grade 1-2 reactive skin capillary hyperplasia occured; Grade 3 AEs occurred in 7 patients (26%); Surgery related complications =14.8%
AVANA (Phase II)	2021	Italy	96 (MSI-H:1; MSS:38; Unknown:57)	Rectal cancer	II-III	nCRT(50.4Gy+Cape) + Avelumab 10 mg/kg q2w → Surgery(8~12 weeks after radiotherapy)	MPR=61.5%(59/96); pCR=23%(22/96)	Grade 3 ~ 4 non-immune and immune related AEs: 8% and 4%, respectively

non-mCRC, Non-metastatic colorectal cancer; nIT, Neoadjuvant immunotherapy; nCRT, Neoadjuvant chemoradiotherapy; SCRT, Short-Course Radiotherapy; dMMR, Deficient mismatch repair; pMMR, Proficient mismatch repair; MSI-H, Microsatellite instability-high; MSS, Microsatellite stability; MPR, Major pathologic response; TRG, Tumor regression grade; pCR, Pathological complete response; cCR, Clinical complete response; W&W, Watch & wait; NAR, Neoadjuvant rectal score; AEs, Adverse effects.

### NICHE Study: “Pioneering Research” of nIT

The single-arm NICHE study from the Netherlands was published in Nature Medicine during 2020 (38). This study examined 40 patients (21 patients with dMMR status and 19 with pMMR status) who had stage I~III colon cancer. The double immune neoadjuvant therapy consisted of nivolumab (anti-PD-1 antibody) combined with ipilimumab (anti-CTLA-4 antibody). Remarkably, all patients in the dMMR group survived without disease, with a median follow-up time of 8.1 months. These results indicated that the nivolumab + ipilimumab regimen was a suitable nIT for patients with dMMR non-mCRC, and suggested that immunotherapy could provide a lasting curative effect after the initial benefit. Moreover, the nIT was safe, feasible, and well tolerated (**Table 1**). Thus, it is likely that nIT would not adversely affect the outcome of the subsequent operation, in that there should be no unexpected or redundant post-surgical complications. The most likely reason for the significant reduction in toxicity was the use of a lower dose of ipilimumab and the shorter duration of the nIT. The NICHE study pioneered the use of nIT for CRC, and therefore provided hope to patients with MSI-H/dMMR LACRC.

### NICOLE Study: “First Exploration” of Monoimmunotherapy

The NICOLE study (NCT04123925) is the first study to examine the effect of neoadjuvant nivolumab for treatment of early-stage colon cancer without selection for MMR status. This study enrolled 44 patients with cT3/T4 resectable colon cancer. The experimental cohort (86% with MSS) received 2 cycles of nivolumab, and then surgery, and the control cohort (77% with MSS) received surgery. The primary end points were the feasibility of preoperative nIT, the degree of pathological response, and molecular and immunophenotypic changes in the tumor and peripheral blood. The results showed that more than 70% of the patients in nivolumab group experienced significant tumor regression (**Table 1**). The nivolumab group also had significantly higher levels of CD8− and CD8+ non-inhibitory T cells.

### VOLTAGE-A Study: “Innovative Exploration” of Sequential nIT With nCRT

The sort-term results of the exploratory VOLTAGE-A phase II study from Japan were published in the Journal of Clinical Oncology during 2020 (39). This study, which compared LACRC patients in an MSS group and an MSI-H group, examined the effect of an initial long-term nCRT, followed by nIT, surgical resection, and then adjuvant chemotherapy. Both groups achieved major pathologic response (MPR) (**Table 1**). As of January 2020, the median follow-up time was 22.5 months for the MSS group and 6.6 months for the MSI-H group. In the MSS group, 2 patients had local recurrence and 2 had distant metastases; however, no patients in the MSI-H group had recurrence.

The FOWARC study (40, 41) found that the pCR rate of mFOLFOX6 combined with preoperative radiotherapy was 28%. However, this improvement of pCR was not accompanied by improvements in final survival. The pCR rate in the VOLTAGE study was 30%, however, whether this treatment can also provide a survival benefit likely depends on whether the nIT after nCRT was able to activate the immune system and remove small residual lesions. A long-term follow-up of patient survival is needed. Although the sample size of the VOLTAGE study was small, it was the first to compare the application of nIT combined with nCRT for patients who had MSI-H and MSS LACRC. Moreover, the results suggested a better combination biomarker for predicting the efficacy of nIT — PD-L1 positivity and a high ratio of CD8+/Treg cells. This combination biomarker has potential for use in subsequent studies.

### NRG-GI002 Study: “Future Prospects” of TNT Combined With nIT

NRG-GI002 is an ongoing phase II clinical research platform that is examining the effect of total neoadjuvant therapy (TNT) for LARC (42). One experimental arm received pembrolizumab with TNT, and researchers evaluated the efficacy and safety of this regimen. The most recent data were presented orally at the 2021 ASCO-GI Annual Meeting (**Table 1**). The results indicated that a TNT consisting of the combination of pembrolizumab with nCRT was safe and led no unexpected short-term toxicities. Nevertheless, it failed to improve the neoadjuvant rectal (NAR) score. In contrast to the previous VOLTAGE-A study of sequential immunotherapy after radiotherapy, this study combined radiotherapy and chemotherapy with immunotherapy. It is not yet known whether this combined therapy will further enhance the sensitivity to radiotherapy or activate the immune response in combination with ICIs while releasing new antigens during radiotherapy. We look forward to seeing the upcoming reports of survival data.

### NCT04231552 Study: “Perfect Combination” of Short-Course Radiotherapy, Chemotherapy, and nIT

Professor Zhang Tao reported a phase II study of delayed surgical treatment of LARC after short-range radiotherapy combined with CAPOX and camrelizumab (anti-PD-1 antibody) at the 2021 ASCO-GI Annual Meeting (43). The results showed that all patients exhibited a very high pCR rate regardless of MMR status (**Table 1**). This study was the first to put forward a new neoadjuvant treatment mode of short-range large-fractionated radiotherapy combined with chemotherapy and a PD-1 inhibitor for patients with LARC. This regimen greatly reduced the preoperative treatment time and cost and ensured a good quality-of-life, and therefore provides a foundation for the formulation of novel neoadjuvant treatment strategies for LARC. These results provide hope to patients with low or very low LARC to achieve organ retention and adopt a “Watch & Wait” (W&W) strategy in the future.

### AVANA Study: “Continuous Exploration” of nCRT Combined With nIT

The AVANA study is a multicenter phase II study that is examining the efficacy of nCRT combined with avelumab (anti-PD-L1 antibody) for treatment of LARC (44). The results suggest that nCRT can induce the antigen release of low neoantigen burden tumors (such as mismatched CRC) and activate dendritic cells, leading to a CD8+ T lymphocyte-mediated anticancer immune response. nCRT combined with avelumab further increased the expression of PD-L1 in tumor cells, so the researchers strongly recommended using the combination of nCRT and ICIs. Among the 96 patients for whom a pathological response was evaluable, 22 (23%) achieved pCR and 59 (61.5%) achieved MRP (**Table 1**).

These six studies illustrate that MSI-H/dMMR LACRC is inseparable from anti-PD-(L)1 neoadjuvant therapy. The exploration of different and novel combinations and targets, such as chemoimmunotherapy, immunoradiotherapy, combinational checkpoint immunotherapy, and even chemoimmunoradiotherapy, can provide new directions for examining the most suitable population of CRC patients, and even patients with other cancers. In addition, we summarized the ongoing clinical trials of nIT for LACRC (**Table 2**).

**Table 2:** Ongoing clinical trials of nIT for LACRC.

NCT number	Study Title	Phase of trial	Number of participants	Condition	Intervention	Primary outcome measures	Secondary outcome measures	Trial status
NCT04130854	INNATE: Immunotherapy During Neoadjuvant Therapy for Rectal Cancer, a Phase II Randomized Multi-center Trial of Neoadjuvant Therapy With and Without APX005M, an Anti-CD40 Agonist, APX005M, for Locally Advanced Rectal Adenocarcinoma	II	58	LARC	Experimental: APX005M(an anti-CD40 agonist)on day 3 of SCRT(5Gy x 5 days) and on day 3 of cycles 1-5 of mFOLFOX → the 6th cycle of only mFOLFOX → Surgery; Active Comparator: SCRT(5Gy x 5 days) + 6 cycle of mFOLFOX →Surgery	pCR rate	OS DFS Rate of resection Safety and tolerability Disease recurrence Development of disease Clinical imaging response cCR rate	Recruiting
NCT04165772	A Phase II Study of Induction PD-1 Blockade in Subjects With Locally Advanced Mismatch Repair Deficient Rectal Adenocarcinoma	II	30	MSI-H/dMMR LARC	9 cycles of TSR-042 (a PD-1 antibody, 500mg; q3w); participants who exhibit CR will proceed to W&W; participants who do not have a CR will received standard nCRT →Surgery	pCR rate; cCR rate	/	Recruiting
NCT04304209	PD-1 Antibody Sintilimab ± Chemoradiotherapy for Locally Advanced Rectal Cancer	II/III	25	LARC (cohort A:dMMR/MSI-H; Cohort B:pMMR/MSS MSI-L;)	Cohort A: 4 cycles of Sintilimab(a PD-1 antibody) → Surgery or W&W → 4 cycles of Sintilimab ± CapeOx according to pathologic response; Cohort B-arm 1: 4 cycles of Sintilimab, nCRT with CapeOx → Surgery or W&W, → 4 cycles of Cape0x; Cohort B-arm 2: 4 cycles of nCRT with CapeOx → Surgery or W&W → 4 cycles of CapeOx	pCR rate	Acute toxicities TRG R0 resection rate Surgical complication Local recurrence Distant metastasis 3-year DFS	Recruiting
NCT03985891	A Randomized, Prospective Clinical Trial of Safety and Efficacy of JS001 Combined With Chemotherapy in Patients With Locally Advanced Colon Cancer (Perioperative Treatment)	I/II	40	LACC	Experimental: JS001(a PD-1 antibody) in combination with FOLFOX for 6 cycles before and after operation. Active Comparator: FOLFOX for 6 cycles before and after operation.	pCR rate; cCR rate; ORR	DFS OS	Recruiting
NCT03299660	Phase II Trial PD-L1/PD-1 Blockade Avelumab (MSB0010718C) With Chemoradiotherapy for Locally Advanced Resectable Rectal Cancer	II	45	LARC	nCRT with capecitabine for 6 weeks → 4 cycles of Avelumab(a PD-L1 antibody) → Surgery	pCR rate	Response as per structural imaging Overall FDG PET response Toxicity Rate of downstaging	Recruiting
NCT04340401	A Phase II Study of Total Neoadjuvant Therapy Plus SHR1210 for High-risk Locally Advanced Rectal Cancer and Biomarker Screening Base on Neoantigen	II	25	LARC	3 cycles Capeox+SHR-1210(an anti-PD-1 inhibitor) → nCRT with capecitabine → 2 cycles CapeOx → Surgery	pCR rate	Toxicity of TNT+SHR-1210 Change of TCR repertoire DFS Surgical complication rate Major AEs	Recruiting
NCT04230759	Radiochemotherapy +/- Durvalumab for Locally-advanced Anal Carcinoma. A Multicenter, Randomized, Phase II Trial of the German Anal Cancer Study Group (RADIANCE)	II	178	LASCC	Experimental: 5FU+Mitomycin C-based radiochemotherapy+Durvalumab (a PD-L1 antibody); Active Comparator: 5FU+Mitomycin C -based Radiochemotherapy	DFS	Major AEs cCR OS Colostomy-free survival locoregional recurrence Distant recurrence Quality of life questionnaires	Recruiting
NCT04293419	Phase II Study of Durvalumab (MEDI4736) Plus Total Neoadjuvant Therapy (TNT) in Locally Advanced Rectal Cancer (The DUREC Trial)	II	58	LARC	6 cycles of mFOLFOX6 → nCRT with capecitabine → Surgery. Patients will receive durvalumab(a PD-L1 antibody,1500mg;q4w) during induction chemotherapy, nCRT and waiting period until surgery	pCR rate	Tumor downstaging TRG R0 resection rate 3-year DFS Toxicity profile Surgical complications NAR score	Recruiting
NCT03127007	A Phase Ib/II Study to Evaluate Safety and Efficacy of Atezolizumab Combined With Radio-chemotherapy in a Preoperative Setting for Patients With Localized Rectal Cancer (R-IMMUNE)	I/II	54	LARC	Experimental: nCRT with 5-FU, Atezolizumab (a PD-L1 antibody, 1200mg) is given on day 1 of week 3, 6, 9 and 12. Surgery is planned during week 15; Active Comparator: nCRT with 5-FU. Surgery is planned during week 15	Rate of AEs; pCR rate	/	Recruiting
NCT05202314	Camrelizumab Combined With Neoadjuvant Chemotherapy After Stent Placement for Left-Sided Obstructive Colonic Cancer (NACSOC-02)	II	20	Obstructive colonic cancer	Camrelizumab (a PD-1 antibody, 200mg) 2 cycles + mFOLFOX6 3 cycles or CapeOx 2 cycles → Surgery	pCR rate	3-year OS 3-year DFS	Recruiting

LARC, Locally advanced rectal cancer; LACC, Locally advanced colon cancer; LASCC, locally-advanced anal squamous cell carcinoma; nIT, Neoadjuvant immunotherapy; nCRT, Neoadjuvant chemoradiotherapy; SCRT, Short-Course Radiotherapy; TNT, Total Neoadjuvant Therapy; dMMR, Deficient mismatch repair; pMMR, Proficient mismatch repair; MSI-H, Microsatellite instability-high; MSI-L, Microsatellite instability-low; MSS, Microsatellite stability; ORR,Objective response rate; TRG, Tumor regression grade; CR, Complete response; pCR, Pathological complete response; cCR, Clinical complete response; W&W, Watch & wait; NAR, Neoadjuvant rectal score; AEs, Adverse effects; OS, Overall survival; DFS, Disease free survival; TCR, T Cell Receptor.

## Retrospective Studies of nIT for MSI-H/dMMR LACRC

In recent years, several retrospective studies performed in diverse countries have examined the effect of nIT for MSI-H/dMMR CRC (**Table 3**).

Table 3Retrospective studies of nIT for MSI-H/dMMR LACRC.

**Table:** 

Published journal	Published date	Country	Cases	Gender	Age (years)	Personal or familial history of cancer	Lynch syndrome	Tumor location	Distance from anal verge (cm)	cTNM stage	MRF	EMVI	Type of pathology	MMR (IHC)	MSI (NGS)	RAS
OncoImmunology	2019.9	China	2	Male	27	Mother:rectal cancer and endometrial carcinoma;mother’s brother:colon cancer	Yes	Rectum	6.4	cT4bN2M0,III	+	+	Adenocarcinoma	MSH2(-), MSH6(-)	MSI-H	Wild type
				Female	64	20 years ago: rectal cancer surgery; 9 years ago: endometrial carcinoma surgery	Yes	Rectum	1	cT4bN2M0,III	−	+	Adenocarcinoma	MSH2(-), MSH6(-)	MSI-H	KRAS Mutation (p.G12D)
EJC	2020.4	Belgium	1	Female	27	Father, paternal grandfather and paternal great-grandfather: colon cancer	Yes	Rectum	N/A	cT3N2 M0, III	+	−	Adenocarcinoma	MSH2(-)	MSI-H	N/A
EJSO	2020.6	China	2	Male	60	N/A	N/A	Descending colon	N/A	cT4aN2M0,III	N/A	N/A	Adenocarcinoma	MSH6 (-)	MSI-H	Wild type
				Female	46	N/A	N/A	Rectosigmoid junctions	N/A	cT4aN2M0,III	N/A	N/A	Adenocarcinoma	MSH2 (-), MSH6 (-)	MSI-H	KRAS Mutation (p.G12D)
JNCCN	2020.7	America	3	Male	81	10 years ago: prostate cancer surgery	N/A	Mid -rectum; lower rectum	5;2	cT3N1M0,III; cT2N0 M0,I	+;−	−;−	Adenocarcinoma	MSH2 (-), MSH6 (-)	MSI-H	N/A
				Male	55	25 years ago: right-sided colon surgery	N/A	Lower rectum	3	cT3N1M0,III	+	−	Adenocarcinoma	PMS2(-)	MSI-H	Wild type
				Female	38	N/A	Yes	Mid -rectum	N/A	cT3N2M0,III	+	−	Mucinous adenocarcinoma	MSH2 (-)	MSI-H	N/A
OncoImmunology	2020.12	China	4	Female	51	N/A	Yes	N/A	N/A	cT3N1M0,III	N/A	N/A	N/A	N/A	MSI-H	N/A
				Female	19	N/A	Yes	N/A	N/A	cT3N1M0,III	N/A	N/A	N/A	N/A	MSI-H	KRAS Mutation
				Female	49	N/A	Yes	N/A	N/A	cT3N1M0,III	N/A	N/A	N/A	N/A	MSI-H	KRAS Mutation
				Male	34	N/A	Yes	N/A	N/A	cT4bN2M0, III	N/A	N/A	N/A	N/A	MSI-H	Wild type

**Table 3:** 

Published journal	Cases	BRAF	TMB	Previous treatment	Previous treatment response	nIT strategy	nIT response evaluation	AEs during nIT	Radical resection after nIT	ypTNM stage	Adjuvant therapy	Follow-up
OncoImmunology	2	Wild type	High	None	None	Nivolumab 3mg/kg,q2w; 6cycles	PR	Fatigue, grade 2	Yes	ypT0N0M0 (pCR)	None	NED (>1 year after operation)
		Wild type	High	FOLFOXIRI;4cycles	SD	Nivolumab 3mg/kg,q2w; 6cycles	CR	None	No	N/A	None	NED (1 year after nIT)
EJC	1	N/A	N/A	None	None	Ipilimumab 1mg/kg + Nivolumab 3mg/kg,q2w; 2cycles; Nivolumab3mg/kg,1cycle	CR	None	Yes	ypT0N0M0(pCR)	nivolumab 3mg/kg, q2w ,4 months	NED (6 months after operation)
EJSO	2	Wild type	N/A	None	None	Toripalimab 240mg +XELOX q3w;4cycles	PR	Nausea, grade 1	Yes	ypT0N0M0(pCR)	None	NED
		Wild type	N/A	XELOX	None	Sintilimab200mg,q3w; 5cycles + bevacizumab 500mg,q3w;4cycles	PR	None	Yes	ypT0N0M0(pCR)	None	NED
JNCCN	3	N/A	N/A	None	None	Pembrolizumab 200mg, q3w;11cycles	CR	Fatigue, grade 2	No	N/A	None	NED (17 months after nIT)
		Wild type	High	FOLFOX; 8cycles	SD	Nivolumab 3mg/kg + Ipilimumab 1mg/kg, q3w;7cycles	CR	Fatigue and rash,grade 1	No	N/A	None	NED (12 months after nIT)
		N/A	N/A	None	None	Pembrolizumab +FOLFOX;7cycles	PR	Rash, grade 1	Yes	ypT0N0M0(pCR)	None	NED (10 months after nIT)
OncoImmunology	4	N/A	N/A	None	None	Pembrolizumab240mg +XELOX,q3w;2cycles	PR	N/A	Yes	ypT0N0M0(pCR)	N/A	N/A
		Wild type	N/A	None	None	Pembrolizumab 200mg +Ipilimumab 50mg,q3w; 4cycles	CR	N/A	No	N/A	N/A	N/A
		Wild type	N/A	None	None	Nivolumab 140mg,q3w; 12cycles	PR	N/A	Yes	ypT0N0M0(pCR)	N/A	N/A
		Wild type	N/A	None	None	Pembrolizumab 200mg,q3w;4cycles	PR	N/A	Yes	TRG 2 (PR)	N/A	N/A

LACRC, Locally advanced colorectal cancer; nIT, Neoadjuvant immunotherapy; nCRT, Neoadjuvant chemoradiotherapy; EJC, European Journal of Cancer; EJSO, European Journal of Surgical Oncology; JNCCN, Journal of the National Comprehensive Cancer Network; MRF, Mesorectal fascia; EMVI, Extramural vascular invasion; MMR, Mismatch repair gene; IHC, Immunohistochemistry; MSI, Microsatellite instability; NGS, Next generation sequencing; TMB, Tumor mutation burden; PR, Partial response; PD, Progressive disease; SD, Stable disease; pCR, Pathological complete response; cCR, Clinical complete response; NED, No Evidence of Disease; TRG, Tumor regression grade; AEs, Adverse effects; N/A, not available;+, Positive;−, Negative.

In September 2019, Chinese researchers first reported that two patients with MSI-H/dMMR LARC received nIT treatment with nivolumab (6 cycles). One patient with low rectal cancer adopted a W&W anus-preserving strategy after achieving cCR, and experienced no recurrence after 1 year of follow-up. The other patient received TME surgery and was confirmed as pCR (45). This study was the first to examine the efficacy of nIT as a treatment for MSI-H LARC. In April 2020, Belgian researchers reported that a patient with MSI-H/dMMR low LARC received nIT consisting of nivolumab + ipilimumab. After 3 cycles, TME surgery was performed and it was pathologically confirmed as pCR (46). In June 2020, Chinese researchers reported that two patients with MSI-H LACRC received nIT alone or in combination with targeted therapy. Both patients had confirmed pCR after the operation (47). In July 2020, researchers from the U.S. reported that three patients with MSI-H/dMMR LACRC received nIT: one with monoimmunotherapy, one with monoimmunotherapy combined chemotherapy, and one with double immunotherapy. Their outcomes were pCR, pCR, and W&W with cCR, respectively (48). In December 2020, a retrospective study examined eight patients with MSI-H locally advanced/recurrent metastatic CRC who received monoimmunization or double immunization. The remission rate was 100%. Among the four LACRC patients, one with cCR adopted a W&W approach, and two had confirmed pCR based on postoperative pathology (49).

## nIT for MSI-H/dMMR LACRC at Our Center

**Figure 2:**
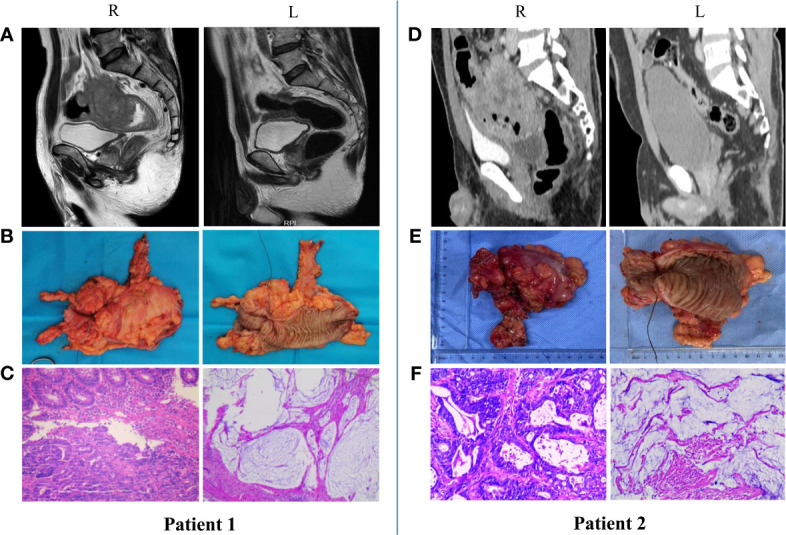
Radiologic and pathological response to nIT with Tislelizumab, and surgical specimens in two patients with MSI-H/dMMR LACRC (pCR). **(A, D)** Imaging: Pretreatment (R) VS After 6 cycles of nIT (L); **(B, E)** Surgical specimens: After 6 cycles of nIT (R/L); **(C, F)** Pathology: Pretreatment (R) VS After 6 cycles of nIT (L).

**Figure 3:**
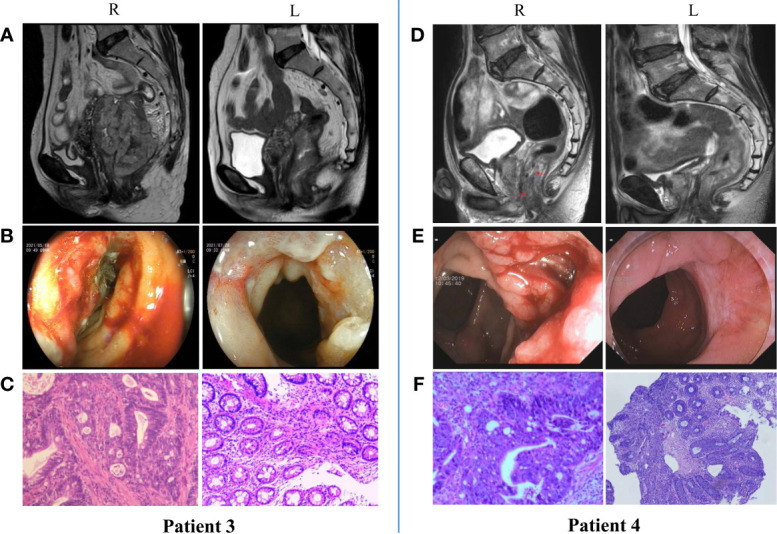
Radiologic, colonoscopic and pathological response to nIT with Tislelizumab in two patients with MSI-H/dMMR LARC (cCR). **(A, D)** Imaging: Pretreatment (R) VS After 6 cycles of nIT (L); **(B, E)** Colonoscopy: Pretreatment (R) VS After 6 cycles of nIT with (L); **(C, F)** Pathology: Pretreatment tumor biospy (R) VS Re-biospy after 6 cycles of nIT (L).

Our center also conducted a trial to examine the efficacy and safety of neoadjuvant anti-PD-1 monoimmunotherapy for patients with MSI-H/dMMR LACRC. At present, 18 patients with MSI-H/dMMR LACRC (including ascending colon cancer, descending colon cancer, sigmoid colon cancer, and rectal cancer) have received nIT with Tislelizumab, and the tumors of all patients (100%) have achieved significant regression. Two patients with LARC and 5 patients with LACC underwent surgery, and all had confirmed pCR (**Figure 2**). Two patients with low LARC adopted a W&W approach after achieving cCR (**Figure 3**). The remaining patients will also undergo surgery. We look forward to reporting the final outcomes, including rates of tumor regression grade (TRG), MPR (TRG 0~1), and pCR (TRG 0).

## New Organ Preservation Strategy for MSI-H/dMMR Low LARC

The W&W non-surgical organ preservation strategy was first reported by Harba-Gamal et al. in 2004 (50). This approach was applied to patients with rectal cancer who had cCR after nCRT. Instead of receiving traditional surgery, these patients entered a period of close follow-up and observation in an effort to preserve organ function without affecting tumor survival rate. There is increasing use of neoadjuvant therapy for rectal cancer, but it must be confirmed whether other patients who achieve cCR after neoadjuvant therapy are suitable for a W&W strategy. However, based on the results of previous studies and the known lasting benefits of immunotherapy that has been effective, patients with cCR after nIT for LARC seem especially well-suited for the W&W strategy.

The breakthrough efficacy of nIT for many patients with MSI-H/dMMR LACRC provides colorectal oncologists with great hope, especially for patients with MSI-H/dMMR low LARC. Patients with low rectal cancer face potential risks from surgery that include anal dysfunction, abnormal fecal control, and sexual dysfunction. The prospective and retrospective studies described above indicate that nIT was associated with less risk of sphincter dysfunction, sexual dysfunction, and bladder dysfunction than traditional nCT and nCRT. Furthermore, nIT can provide lasting benefits to the patient. By adopting a W&W strategy after a patient with MSI-H/dMMR low LARC achieves cCR due to nIT, surgery and its concomitant complications can be avoided, thus providing organ preservation and improving the long-term prognosis. It remains very important to develop additional biomarkers for complete remission as future developments of nIT continue.

## Conclusions and Perspectives

In summary, we suggest that careful selection of neoadjuvant therapy for patients with LACRC should be guided by risk degree classification and molecular typing. Thus, patients with MSI-H/dMMR LACRC typically have poor sensitivity to traditional nCRT and nCT. A nIT that uses a single ICI, or two ICIs, or an ICI combined with nCRT/nCT can be considered as a reasonable choice and promising strategy for these patients.

The use of molecular screening to identify patients most suitable for specific nITs will allow clinicians to formulate more reasonable individualized layered diagnoses and treatment strategies for patients with LACRC. This approach may help prevent poor long-term prognosis caused by treatment deviation or deficiency, and may prevent the decline in quality-of-life caused by over-treatment. For patients with low MSI-H/dMMR LARC, a W&W strategy after achieving cCR following nIT could provide opportunities for organ preservation without adversely affecting prognostic survival. Further studies of nIT for LACRC should consider relevant biomarkers in combination with TMB, TNB, liquid biopsy assays, TME immune scores, and other methods to develop a predictive system or model that improves prognosis and clinical efficacy from multiple dimensions.

## Author Contributions

Drafting the work and/or revising it critically: XZ, TW, and XC.Final approval of the version to be published: XZ, and YL. All authors contributed to the article and approved the submitted version.

## Funding

This study was supported by the National Natural Science Foundation of China (82060542 to CD), and the Yunnan Provincial Department of Education Science Research Fund Project (2022J0227 to XZ).

## Conflict of Interest

The authors declare that the research was conducted in the absence of any commercial or financial relationships that could be construed as a potential conflict of interest.

## Publisher’s Note

All claims expressed in this article are solely those of the authors and do not necessarily represent those of their affiliated organizations, or those of the publisher, the editors and the reviewers. Any product that may be evaluated in this article, or claim that may be made by its manufacturer, is not guaranteed or endorsed by the publisher.
